# Classification of Actigraphy Records from Bipolar Disorder Patients Using Slope Entropy: A Feasibility Study

**DOI:** 10.3390/e22111243

**Published:** 2020-11-01

**Authors:** David Cuesta-Frau, Jakub Schneider, Eduard Bakštein, Pavel Vostatek, Filip Spaniel, Daniel Novák

**Affiliations:** 1 Technological Institute of Informatics, Alcoi Campus, Universitat Politècnica de València, 46022 Valencia, Spain; 2 Department of Cybernetics, Czech Technical University in Prague, 166 36 Prague, Czech Republic; schnejak@fel.cvut.cz (J.S.); eda@zzz.cz (E.B.); xnovakd1@fel.cvut.cz (D.N.); 3 National Institute of Mental Health, 250 67 Klecany, Czech Republic; Filip.Spaniel@nudz.cz; 4 MINDPAX, Vinohrady, 128 00 Prague, Czech Republic; voo@centrum.cz

**Keywords:** bipolar disorder, actigraphy, sample entropy, permutation entropy, slope entropy, time series classification

## Abstract

Bipolar Disorder (BD) is an illness with high prevalence and a huge social and economic impact. It is recurrent, with a long-term evolution in most cases. Early treatment and continuous monitoring have proven to be very effective in mitigating the causes and consequences of BD. However, no tools are currently available for a massive and semi-automatic BD patient monitoring and control. Taking advantage of recent technological developments in the field of wearables, this paper studies the feasibility of a BD episodes classification analysis while using entropy measures, an approach successfully applied in a myriad of other physiological frameworks. This is a very difficult task, since actigraphy records are highly non-stationary and corrupted with artifacts (no activity). The method devised uses a preprocessing stage to extract epochs of activity, and then applies a quantification measure, Slope Entropy, recently proposed, which outperforms the most common entropy measures used in biomedical time series. The results confirm the feasibility of the approach proposed, since the three states that are involved in BD, depression, mania, and remission, can be significantly distinguished.

## 1. Introduction

Bipolar Disorder (BD) is a chronic mental illness with a prevalence of approximately 1–2% [1,2]. It has high heritability rate and equal distribution across both genders [3]. The main symptom is the recurrent changing of symptomatic episodes of depression or of elevated mood (mania) with non-symptomatic (remission) periods [4]. The factors contributing to relapse in BD are not yet clearly understood, but it has been suggested that there is an association with the dysregulation of circadian rhythm and sleep [5,6].

There have been many attempts to rate or quantify the level of depression or mania [7]. The ultimate goal is to evaluate the effects of treatment. Most of these approaches are based on a set of psychiatric symptoms, as those described in the comprehensive study [8]. In this work, the 17 most commonly found symptoms in depression were first identified. Subsequently, a depression scale was created using 10 out of these 17 symptoms, those that exhibited the largest variation with treatment, and the highest correlation with changes. A similar rating approach could be applied to BD, but, in the case of BD, it is more important at first to distinguish between the three mood states. Such technical approach could be more convenient in this case than a classical psychiatric approach.

The three possible episodes: depression (dep), mania (man), and remission (rem) are hypothesized to be linked to different degrees and patterns of physical activity [9]. Taking advantage of all the disparity of wearables available nowadays, with actigraphy monitoring and recording capabilities, it is reasonable to assume that a suitable analysis of the resulting actigraphy time series could become a reliable tool for diagnosis and assessment in BD.

An actigraph is a wrist-worn device used for inexpensive evaluation of sleep and circadian rhythms [10,11,12] in common conditions (ambulatory patients mainly). In general, motor activity measurement or actigraphy can be used for a disparity of clinical purposes: to quantify physical activity, in chronobiology applications, to detect sleep patterns and stages, and many more that are related to health and diseases [10]. For example, and specifically in the case of BD, the dysregulation of rhythmicity that is connected to BD and the Krane–Gartiser actigraphy study described in [9] suggested that the complexity of activity differs among mood episodes. However, the short duration of the actigraphy follow-up period in most of these studies poses the main challenge in comparing data from symptomatic periods, since they occur quite rarely [2] (once in every two years).

We devised a study to compare actigraphy recordings from in vivo symptomatic mood episodes of outpatients with a long follow-up period (up to two years). Using this new scheme, we were able to explore actigraphy data from remission periods as well as relapse episodes of mania and depression, gaining new insight into disease progression and outcome.

However, manual inspection of these long-term records is cumbersome and error prone. The time series are very noisy and the important information might be scarce and blurred by artifacts or other activity aspects independent of the disease state. Because the use of non-linear methods to expose hidden characteristics of time series has proven to be a very powerful tool in similar frameworks, we studied the feasibility of such methods in this new signal classification task at hand. Recent works have already pointed in that same direction, such as [13]. In this work, 106 bipolar I type patients, 73 unaffected siblings, and 76 control subjects with valid actigraphy and sleep diary data for at least eight days were included in the analysis. This analysis was based on Detrended Fluctuation Analysis [14], using six time windows. The results gave evidence of significant differences between control and bipolar patients, but no differences between depressive or manic symptoms were found in the patients group.

There are many more non-linear signal analysis methods described in the scientific literature. We first explored the most promising methods: Sample Entropy (SampEn) [15], Permutation Entropy (PE) [16], and a few derived methods that apparently yield a better classification performance [17], quantified in terms of accuracy. Some of these methods are Weighted PE (WPE) [18], Bubble Entropy (BE) [19] and Slope Entropy (SlopEn) [20], and they have the theoretical advantage of using more than a single source of information, mainly amplitude and ordinal information. From this exploratory analysis, we chose the final method according to the highest classification performance achieved, in this case SlopEn. This performance has been recently confirmed in another classification study [21].

**Figure 1:**
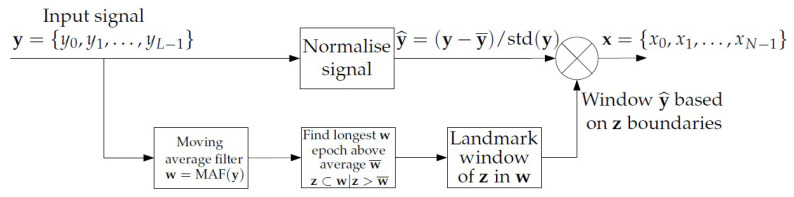
General diagram of the method proposed.

A preprocessing stage was also included in order to improve the quality of the data to be analysed. This preprocessing isolated the epochs of greatest activity and used them for classification purposes based on SlopEn, omitting periods of no-activity (sleep mainly) or with too short activity. Figure 1 shows a general diagram of the proposed method.

According to the achieved results, the method can robustly distinguish between dep and man records, with an accuracy higher than 70% in most cases and, less robustly, yet still statistically significant, for dep–rem and man–rem (61% and 63%, respectively). These promising results arguably entail a new line of research worth exploring, with a lot of room for improvement both in terms of signal acquisition (more stable and longer periods of activity, better separation of activity, and no-activity epochs), and signal processing (more optimised entropy measures, better input parameter settings).

## 2. Materials and Methods

### 2.1. Dataset

Actigraphy data were recorded in 94 BD patients, some of them with several episodes, recruited while using web forms, and collecting basic demographic information and personal history of BD diagnosis. All of the patients, who fulfilled the requirements, were examined by psychiatrists at the National Institute of Mental Health (NIMH), in Klecany, Czech Republic, for confirmation of the BD diagnosis according to the DSM-5 criteria [4]. The duration of the recordings was up to two years. The device used was the MINDPAX, provided by the Mindpax company (http://mindpax.me).

MINDPAX actigraphy wearable uses an internal three-axis accelerometer for measurement, with a sampling frequency of 6 Hz, aggregated into 30-s epochs. The data used for analysis were 14 day long segments from periods of remission, depression, and mania state. The study was approved by the ethical committee of the NIMH, Czech Republic, and all of the BD patients signed written informed consent. On enrolment to the study, after the confirmation of BD diagnosis the patients were equipped with a wrist-worn actigraphy monitoring device (MINDPAX) and were instructed to wear it on their non-dominant hand wrist and remove it only when necessary. They were also asked to fill in a weekly mood questionnaire, while using MINDPAX mobile software application.

We analysed data segments that were chosen in such a way that they contained a minimum (at most 5%) of invalid (missing samples) data points. A final set of 44 dep, 16 man, and 137 rem records were analysed. Multiple episodes can be found for some patients in each set, or episodes of the same patient in more than a set. An experiment using only a record from subject in each class was devised in order to address this possible confounding factor.

**Figure 2:**
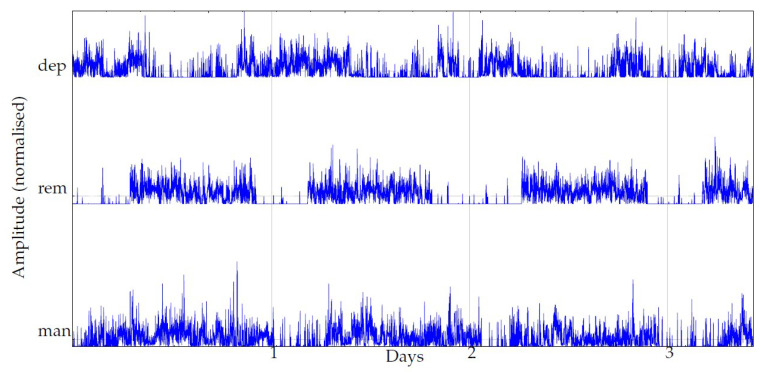
Example of signals from the three classes in the experimental database. The plots shown correspond to the actigraphy data as they were captured with the monitoring device, no filtering or preprocessing yet. One day (24 h) corresponds to 2880 samples (sampling period 30 s).

The state was annotated, by a team of experts, based on a monthly Montgomery–Åsberg Depression Rating Scale (MADRS) [8], the Young Mania Rating Scale (YMRS) [7], weekly self-mood-estimation questionnaires, medical records, and additional information at the same monthly temporal scale. All of the patients in the study were examined monthly by trained psychologists using MADRS and YMRS. The periods from hospitalisations were excluded, due to the restriction of physical activity during hospital care. An example of these records in their raw state is shown in Figure 2.

### 2.2. Preprocessing

**Figure 3:**
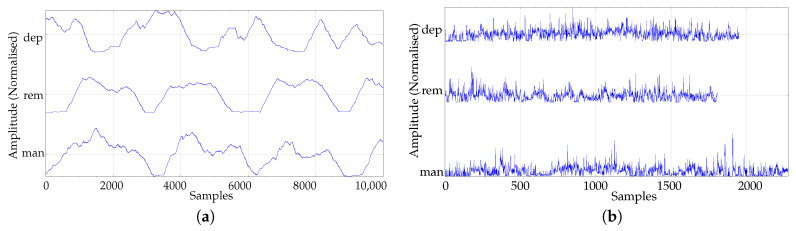
Examples of the prepocessing stage and the result of activity epoch extraction. (**a**) Moving average filtered signal from Figure 2 in order to discriminate between periods of activity and no activity. (**b**) Epochs of activity extracted from each original record according to the threshold applied to the filtered signal. Minimum length obtained was 1000 samples. The activity part was not dependent on the state.

A preprocessing stage was devised to extract periods of high activity and of a substantial duration in order to improve the quality of the possible information provided by these actigraphy records. To this end, signals were first filtered using a moving average filter, window length of 250 samples, with the objective of outlining those periods where the subjects had a significant activity. Then, a thresholding approach was applied to extract those periods from the time series. The threshold employed was the average of the entire filtered signal. Of those epochs above, the threshold, the longest corresponding sequence from the original time series was finally chosen as the representative of the time series. Figure 3 graphically shows the results of this process.

All of the the resulting records had a length of at least 1000 samples. They were normalised before entropy calculation, zero mean, and standard deviation one.

### 2.3. Slope Entropy

SlopEn is a very recently proposed entropy measure [20] that is based on patterns of differences between consecutive samples in time series. With this new approach, the objective is to carry out a gradient pattern analysis instead of an amplitude or ordinal one. We hypothesize that this is an efficient way to characterise variability patterns useful to find possible differences between time series datasets. In fact, several forms of gradient analysis have been previously used successfully for classification [22], and the gradient itself of a time series has proven to be a good information–carrying feature [23]. In addition, coarse–graining strategies are common in similarity searches [24], and in the well known Lempel–Ziv complexity measure [25], since distinguishing features can then become more apparent than using all the information available. On top of that, using more than two quantification states has yielded better results than classical binary approaches in some studies to assess complexity changes in time series [26].

In its seminal study, it outperformed many of the most popular and powerful entropy measures in terms of class discriminating power, while using a very heterogeneous experimental set. For the present paper, given the difficulties of the specific physiological records addressed, we first again explored the most successful entropy measures and the latest developments in this field. Specifically, we tested SampEn [15], PE [16], WPE [18], BE [19], and Slope Entropy. Briefly, these candidate methods were chosen for the following reasons:

- SampEn. It is probably the most frequently applied entropy measure. It was an evolution of Approximate Entropy [27] that solved some of its few problems. It has a high discriminating power even under difficult conditions [28,29,30,31].
- PE. It is getting a lot of attention in the last years due to its simplicity, robustness, and good discriminating power. It has been successfully used in many time series classification studies so far [32,33,34], and many methods have been derived from it [35,36].
- WPE. This is one of the PE derived methods. It includes amplitude information on the PE computation. It has demonstrated a very high discriminating power and stability in a recent comparative study [17].
- BE. This is a very recent method that also improves the performance of PE, with less dependence on input parameters [19]. It has exhibited a good complementarity to PE, with good classification performance in cases where PE was unable to find significant differences [37].
- SlopEn. Recently proposed, it showed a higher discriminating power than PE and WPE [20] for a disparity of records. This was the final choice, since it yielded the best classification performance in an exploratory analysis, as described in Section 3. Therefore, this will be the method described in detail next.

SlopEn is defined, as follows. Given an input time series $x=\left\{x_{0},x_{1},\ldots ,x_{N-1}\right\}$, where $x_{i}$ is the i−th amplitude sample, with *N* samples, and a subsequence of x of length *m* commencing at sample *j*, $x_{j}^{m}=\left\{x_{j},x_{j+1},\ldots ,x_{j+m-1}\right\}$, an associated symbolic pattern to $x_{j}^{m}$ has to be computed. To this purpose, two thresholds have also to be defined: γ, and δ, with γ>δ>0. Thus, if the difference between two consecutive samples of the subsequence is defined as $d=x_{j+1}-x_{j}$, each symbol is:

- 2, if d>γ.
- 1, if d≤γ and d>δ.
- 0, if $\left|d\right|\leq \delta$.
−1, if d<−δ and d≥−γ.
−2, if d<−γ.

Once all of the symbols are computed for a subsequence, the relative frequency of the resulting pattern is updated, while using a dynamic list containing all the different patterns found up to sample *j*. All of the steps are detailed in [20], including numerical examples and C++ source code for SlopEn (Matlab^®^ source code in Appendix A). The numerical values obtained from the Shannon entropy of the relative frequencies can be normalised using a common reference in order to keep the SlopEn range within desired limits (for example, between 0 and 1). The SlopEn result of each record will be the records’ feature to be used in the classification analysis using a threshold, as described in the next section.

### 2.4. Performance Evaluation

The analysis on actigraphy records will be quantified in terms of classification accuracy (percentage of dep, man, and rem records correctly assigned to their class). Given the general problem of classifying objects from two generic classes, A and B, and using the popular definition of True Positive ($T_{P}$), as the outcome when a time series from class A is correctly classified as A, True Negative ($T_{N}$), as the outcome when an instance of class B is correctly classified as B, False Positive ($F_{P}$), when an object of B is classified as A, and False Negative ($F_{N}$), when a record from class A is classified as B, the final parameters that are used for assessment will be: Sensitivity $=\frac{T_{P}}{T_{P}+F_{N}}$, Specificity $=\frac{T_{N}}{T_{N}+F_{P}}$, and Accuracy $=\frac{T_{P}+T_{N}}{T_{P}+F_{P}+T_{N}+F_{N}}$. Statistical significance *p* of the differences between SlopEn values of the two classes under analysis in each experiment was assessed using the Wilcoxon signed-rank hypothesis test, with a significance threshold of p<0.05. Further characterisation of the classification performance using SlopEn was carried out using the Matthews Correlation Coefficient (MCC) [38], a more robust metric when classes are unbalanced. MCC ranges from −1 to 1, with 1 for a perfect classification, 0.5 for 75% correct prediction, and 0 for a random guess [21].

**Figure 4:**
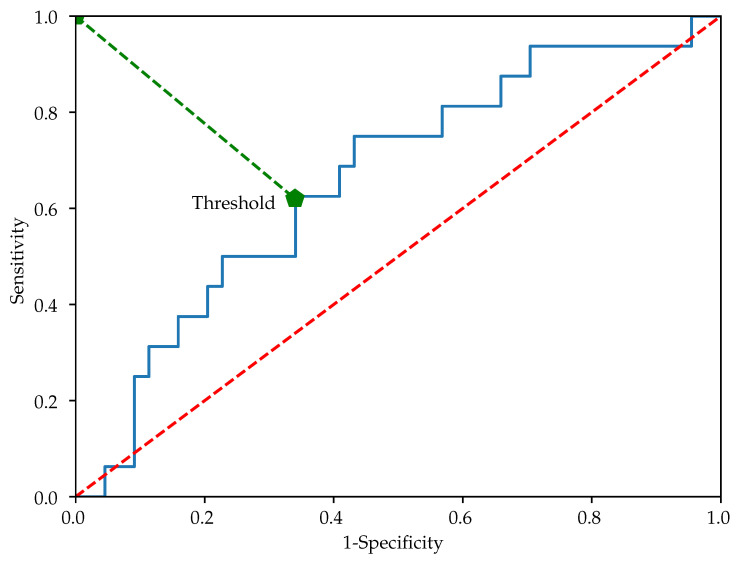
Example of ROC curve obtained in the experiments from which a classification threshold is computed according to the nearest point in curve to (0,1).

Additionally, cross validation will be applied in order to better evaluate the results of the classification. Specifically, the Leave-One-Out (LOO) method, a k−fold cross validation method of size 1 [30], will be applied to the final configuration of the experiments. To this end, in each LOO experiment, a time series from each class will be removed randomly from the experimental dataset used to obtain the classification. From the resulting classification, a SlopEn threshold will be obtained from the associated ROC curve from the nearest point to (0,1) [30], and this threshold will be applied to the removed records. An example is shown in Figure 4. The accuracy in this case will be measured in terms of percentage of correctly classified removed records. A total of 1000 LOO realisations were used in the experiments, with random extraction and replacement (bootstrap).

## 3. Experiments and Results

The first stage of the experiments was an exploratory analysis using several entropy calculation methods in order to choose the approach most likely to be successful in the difficult task of finding significant differences among the three classes available (grid search parameter values), analysed on a two by two basis. Table 1 shows the classification accuracy results of this exploratory analysis.

Because the best performance was achieved using SlopEn, this was the method finally configured for a general classification analysis (statistically significant differences in all comparisons), although, on a single case-by-case basis, there were specific higher accuracies. In order to keep the computational burden of this configuration within reasonable limits, parameters *m* and δ were kept constant, and only γ was varied, from 0.10 up to 0.90, in 0.10 steps. Table 2 depicts the performance achieved, omitting the intermediate γ values that did not provide significant results, until γ=0.80.

Table 3 shows a finer tuning of the SlopEn parameters. Once the region 0.80–0.90 was considered to be the optimal region, since it was the only region with statistically significant results in all cases, γ was varied in 0.01 steps between 0.80 and 0.95 for further optimisation. All of the additional values tested also yielded significant results for the three classification problems addressed. However, the results for γ=0.94 seemed to slightly be above the others, and this was the parameter value finally chosen for the later experiments. For this configuration, and after normalising the SlopEn results by the maximum SlopEn value obtained in all the time series (to keep the values within the 0–1 range [39]), the entropy values for each class were 0.65±0.06 for rem, 0.63±0.06 for dep, and 0.68±0.07 for man. Anyway, any other configuration would have been equally acceptable.

As stated in Section 2.1, the experimental dataset contained, in some cases, more than a single episode per subject and per state, or a subject had episodes in more than a single state. We devoted more experiments to assess the possible influence of these many-to-one and one-to-many correspondences. First, the classification analysis was repeated removing any episode duplication per subject. In this case, the number of objects per class was reduced to 35 for dep, 15 for man, and 77 for rem. Table 4 shows the results.

Subsequently, the classification analysis was repeated removing any subject from the original dataset that only had data in one state (except for man class, due to its small size). In this case, the number of objects per class was reduced to 34 for dep, 16 for man, and 49 for rem. Table 5 shows the results.

For comparative purposes, if this processing was applied using another popular metric in actigraphy records classification tasks [40], the signal mean (before normalisation), the achieved results were 0.74 and 0.75 for Se and Sp, respectively, with p=0.0038 when comparing dep and man records, 0.72 and 0.51, with p=0.0295, for dep and rem, and 0.58 and 0.68, with p=0.1283, for man and rem.

Because the actigraphy records were relatively long, with 40,320 samples, additional experiments were conducted while using more than a single best representative epoch for time series. In the previous results, only the longest epoch was used, which was assumed to feature the most stable and longer activity period of each subject. In order to use more data from the available records, all epochs longer than a predefined *N* threshold were included in the analysis, the same as in Table 3. The tested signals were of lengths N=250 to 2000, with a 250 step. As a consequence, the number of records finally processed, *n*, also varied. Table 6 shows these results.

Given that most entropy quantification methods are length–sensitive, a specific test was devised to find out whether record length played a significant role in the classification performance results. Instead of using the longest epoch available (Table 3), or as many records of a specific length available, as in the previous case (Table 6), in this experiment maximum length records extracted were cut short to 1000 samples in all cases. In other words, a time series of 1000 points was the single representative from each record. Table 7 shows the corresponding results.

Finally, the results of the LOO analysis are shown in Table 8, where, in each experiment realisation, an epoch is randomly left out. These results are more representative of the possible classification performance achievable in a real application using the method proposed.

## 4. Discussion

The initial exploratory analysis was devoted to select the most suited method to the classification of actigraphy records. The candidates corresponded to some of the most widely used entropy methods in similar tasks, whose performance has been assessed in multiple studies. As expected, all of them yielded reasonably good results, after a limited grid search for optimal parameter configuration and avoid over-fitting.

According to the values presented in Table 1, the classification results were the highest for classes (dep,man) using any of the tested methods. Specifically, SampEn achieved the highest performance, with a classification accuracy of 0.80, and the lowest was achieved using WPE, 0.70, but did not reach statistical significance (p=0.0697). The other methods yielded significant results, with a performance of 0.73 for PE, and 0.77 for both BE and SlopEn. It is important to note that, although only the best results were reported in Table 1, these results were very robust in terms of parameter values, with very similar performances for a wide range of input parameters. Therefore, this case, classes (dep,man), can be considered easy to classify while using a diverse set of entropy methods with a small parameter configuration effort.

The classification of classes (dep,rem) was more difficult, although all of the methods exhibited significance in their classifications. The accuracy was lower, 0.67 at most, again for SampEn, but also for PE, with BE and SlopEn slightly behind with 0.65. WPE was again the worst performing method, with only 0.61. It is important to note that input parameters for the methods were, in general, different to those that were used in the previous case.

The last case, the discrimination between classes man and rem, was, by far, the most difficult case. Despite a relatively extensive grid search for parameter values (*m* from 3 to 8, and m=2,3 for SampEn, with *r* from 0.15 up to 0.30), only SlopEn was capable of finding statistically significant results, although with PE that is relatively close (p=0.0579). This is the case that made the difference, with a parameter configuration for SlopEn of m=6, γ=0.85 and $\delta =1\times 10^{-3}$.

Once SlopEn was considered to be the best choice, a finer parameter tuning process was conducted in order to find out if the performance could be improved further. In order to keep the complexity of the process within reasonable limits, tuning was only applied to parameter γ. The main goal of this process was to find a single parameter configuration that could significantly find differences for all the cases studied simultaneously, since that is more practical for real applications. This analysis is summarised in Table 2. It can be observed that, for γ=0.10,…,0.90, the highest significant accuracy corresponds to the region above 0.80.

The final stage of this SlopEn parameter customisation scheme is shown in Table 3. Although the classification results for γ≥0.80 remained quite stable in terms of significance and accuracy, the specific value γ=0.94 was taken as the optimal value to use in subsequent experiments. It is important to be aware of the fact that the grid search was not exhaustive, γ was varied, keeping *m* and δ constant, and that could entail that other better configurations were overlooked. However, from all the parameter regions explored, it can arguably be concluded that no great difference was likely to be found. Moreover, a combination of parameter values, from the best case for each pair of classes, could yield even higher classification results. For example, γ=0.20 yielded an accuracy of 0.77 for classes (dep,man), whereas the chosen value, 0.94, achieved 0.75. Anyway, such an approach would overcomplicate things and it would be more likely to result in data over-fitting. As a consequence, γ=0.94 was the chosen value, keeping in mind that other values could provide slightly better performances. With a different optimal parameter configuration, the results in Table 7 confirmed that time series length did not play a significant role in classification performance.

The results presented in Table 4 and Table 5 were obtained removing duplicities, or subjects featured by a single state. Using the same input parameters as for the entire dataset, the results were reasonably stable. However, statistical significance was not achieved for the classification of man and rem classes. This is the case most difficult to classify, and it seems that a reduction in class objects has a detrimental impact on significance, despite achieving a similar overall classification accuracy. On the other hand, the separation of dep and man classes is fairly robust, with a high accuracy.

The results using more than one epoch per time series exhibited a similar behaviour (Table 6). For relatively few samples (250 samples correspond to 125 min), the number of epochs processed grew significantly (3198 and 2853 respectively), but the performance was poor. It can be hypothesised that few samples do not suffice to reflect the status of the subject in terms of entropy computation. On the other end, if the length of activity required is too large in the preprocessing stage, many records are not represented in the final experimental set, since they do not contain epochs of stable activity (no inactivity periods interspersed) of such length, and therefore the classes become more unbalanced. The length zone around 1000 samples is probably the best one, since at least all of the time series are still represented, and many of them with more than a record. In fact, these are the results closer to those that were achieved using the longest epoch as in Table 3.

The LOO analysis yielded a slightly lower accuracy than the classification using the entire dataset, as expected, since the thresholds were computed with some instances, and applied to the removed instances that did not have anything to do with that computation in order to assess genericity. Despite this reduction in accuracy, the LOO results were arguably very close to their counterparts using all of the records, 0.75 vs. 0.77, 0.58 vs. 0.61, and 0.62 vs. 0.63, for pairs (dep,man), (dep,rem), and (man,rem), respectively. Once more, it is apparent that dep and man records can be easily distinguished, whereas the other two cases will probably need further studies in order to achieve a higher performance.

## 5. Conclusions

Actigraphy is a promising tool for assessing the differences among the episodes that can be found in BD patients. Long term monitoring enabled by advanced wearables pave the way for better analysis, but manual inspection of the resulting records can be difficult and time consuming. Entropy related methods can be successfully introduced in this context to aid in this regard, as the results of this paper have demonstrated.

All of the classification analyses carried out in the present study have demonstrated that it is feasible to discriminate between dep and man episodes fairly easily, with accuracies in the vicinity of 0.75, balanced sensitivity and specificity, and good statistical significance. The other two comparisons, dep–rem and man–rem, are more difficult, with borderline classification results that would need additional classification features, or a finer input parameter tuning.

From a practical perspective, and keeping in mind that further studies are still necessary, the proposed method could be implemented by detecting activity periods of at least 1000 samples (movement above certain predefined threshold), and applying the SlopEn configuration of Table 7, among others, to the data. The resulting value could then be classified as dep, rem, or man, also using a set of predefined thresholds or any other kind of classifier.

The results achieved in the present study could also be further improved while using recent straightforward approaches described in the scientific literature. For example, it can be hypothesised that there is some synergy between methods that could be exploited. This synergy could be exploited when considering each method as a feature of a multidimensional vector, and apply a clustering algorithm to find differences between classes, as in [37], or use each method as the independent variable of a model that assigns a probability to each class [41], among many other pattern recognition methods.

Another possibility is to avoid the information reduction that mapping relative frequencies of SlopEn patterns using Shannon entropy entails. Relevance feature analyses have demonstrated that not all relative frequencies account equally for class differences [42], and that was practically demonstrated in [43]. Future studies should assess the role of each symbolic pattern for SlopEn, or even other similar measures, such as PE, on the differences found among actigraphy records.

Finally, SlopEn still has a long way to go in terms of performance optimisation. The SlopEn configuration used in the present paper is the baseline configuration described in the seminal paper [20]; the only difference is that records were normalised in this case. In addition to the grid search conducted, another optimisation could be to use a non-symmetric approach (use different thresholds for rising and falling slopes), vary also parameter δ, and use a different number of slope thresholds, instead of only two.

## Figures and Tables

**Table 1:** Exploratory analysis results using several entropy methods. Significance related to differences between entropy results from each class. Only SlopEn was able to find statistically significant differences between all the signal classes pairs. It is important to note that accuracy has to be understood in terms of *p*, since groups are unbalanced and a high accuracy can be due to a correct classification of the most populated class, but with a very poor accuracy for the minority class. The significance *p* accounts for this possible variation and the Matthews Correlation Coefficient (MCC) result was also included for SlopEn with the same purpose.

	SampEn	WPE	PE	BE	SlopEn
Classes (dep,man)	Accuracy = 0.80	Accuracy = 0.70	Accuracy = 0.73	Accuracy = 0.77	Accuracy = 0.77
	MCC = 0.4614	MCC = 0.1108	MCC = 0.3735	MCC = 0.2548	MCC = 0.4276
	p=0.0080	p=0.0697	p=0.0059	p=0.0093	p=0.0062
	m=2,r=0.25	m=6	m=5	m=3	$m=6,\gamma =0.20,\delta =1\times 10^{-3}$
Classes (dep,rem)	Accuracy = 0.67	Accuracy = 0.65	Accuracy = 0.67	Accuracy = 0.65	Accuracy = 0.65
	MCC = 0.2535	MCC = 0.2610	MCC = 0.2022	MCC = 0.2475	MCC = 0.2465
	p=0.0025	p=0.0167	p=0.0108	p=0.0015	p=0.0213
	m=3,r=0.25	m=7	m=7	m=3	$m=6,\gamma =0.30,\delta =1\times 10^{-3}$
Classes (man,rem)	Accuracy = 0.68	Accuracy = 0.61	Accuracy = 0.62	Accuracy = 0.61	Accuracy = 0.68
	MCC = 0.1531	MCC = 0.0951	MCC = 0.1323	MCC = 0.2006	MCC = 0.2206
	p=0.2995	p=0.4382	p=0.0579	p=0.1490	p=0.0332
	m=3,r=0.30	m=6	m=7	m=7	$m=6,\gamma =0.85,\delta =1\times 10^{-3}$

**Table 2:** Fine tuning of the γ parameter for SlopEn using a grid search and trying to maximise the performance in terms of classification accuracy linked to statistical significance. Intermediate results (from 0.30 up to 0.80) are not included, because they were not significant for man–rem until γ=0.80 was reached. For all cases m=6 and $\delta =1\times 10^{-3}$.

		γ=0.10	0.20	0.30	0.80	0.90
Classes (dep,man)	Se	0.70	0.81	0.79	0.75	0.68
	Sp	0.69	0.62	0.62	0.69	0.75
	Acc	0.70	0.77	0.75	0.73	0.70
	*p*	0.0089	0.0062	0.0150	0.0053	0.0089
Classes (dep,rem)	Se	0.57	0.61	0.54	0.63	0.61
	Sp	0.67	0.58	0.68	0.59	0.59
	Acc	0.64	0.59	0.65	0.60	0.60
	*p*	0.0099	0.0098	0.0213	0.0357	0.0334
Classes (man,rem)	Se	0.59	0.74	0.73	0.79	0.65
	Sp	0.69	0.62	0.62	0.56	0.62
	Acc	0.68	0.63	0.63	0.58	0.62
	*p*	0.0915	0.0680	0.1284	0.0385	0.0420

**Table 3:** Results for a final fine tuning of the γ parameter value for SlopEn. The optimal value was found to be 0.94, as highlighted in the corresponding column. For all cases m=6 and $\delta =1\times 10^{-3}$.

		γ=0.85	0.86	0.94	0.95
Classes (dep,man)	Se	0.75	0.73	0.75	0.73
	Sp	0.69	0.69	0.75	0.75
	Acc	0.73	0.72	0.75	0.73
	*p*	0.0055	0.0084	0.0077	0.0059
Classes (dep,rem)	Se	0.63	0.61	0.66	0.63
	Sp	0.57	0.59	0.60	0.60
	Acc	0.58	0.60	0.61	0.61
	*p*	0.0419	0.0457	0.0221	0.0262
Classes (man,rem)	Se	0.65	0.65	0.71	0.67
	Sp	0.69	0.69	0.62	0.62
	Acc	0.68	0.68	0.63	0.63
	*p*	0.0332	0.0358	0.0358	0.0379

**Table 4:** Classification accuracy achieved removing duplicated states per subject.

	Classes (dep,man)	Classes (dep,rem)	Classes (man,rem)
Se	0.74	0.65	0.66
Sp	0.73	0.61	0.60
Acc	0.74	0.62	0.61
*p*	0.0132	0.0200	0.1266

**Table 5:** Classification accuracy achieved removing subjects with data only in one state (except for the man class).

	Classes (dep,man)	Classes (dep,rem)	Classes (man,rem)
Se	0.76	0.64	0.69
Sp	0.75	0.69	0.62
Acc	0.76	0.67	0.64
*p*	0.0055	0.0032	0.1316

**Table 6:** The results obtained using all epochs found in the experimental set with lengths 250,500,750,1000,1250,1500,1750 and 2000, instead of only the longest one. At least one representative epoch was found in this experiment for up to length N=1000 from each record, whereas it was possible to draw more than one epoch in other records (That is why n=3198 for N=250, but only n=149 for N=2000, some records were not included in the experiment in that case). Each column refers to records of the same length, as shown in the *N* row. For all cases m=6, γ=0.94 and $\delta =1\times 10^{-3}$. In a few cases Acc coincides with Sp or Se due to value rounding and classes imbalance.

	*N*	250	500	750	1000	1250	1500	1750	2000
	*n*	3198	2853	2449	2119	1846	1397	668	149
Classes (dep,man)	Se	0.68	0.73	0.68	0.67	0.68	0.67	0.74	0.81
	Sp	0.54	0.55	0.64	0.70	0.67	0.69	0.61	0.60
	Acc	0.64	0.68	0.67	0.68	0.68	0.67	0.70	0.75
	*p*	0.0001	0.0001	0.0001	0.0001	0.0001	0.0001	0.0001	0.0268
Classes (dep,rem)	Se	0.54	0.55	0.58	0.53	0.61	0.63	0.64	0.65
	Sp	0.53	0.54	0.54	0.62	0.56	0.58	0.57	0.63
	Acc	0.53	0.55	0.55	0.60	0.57	0.59	0.59	0.64
	*p*	0.0014	0.0001	0.0001	0.0001	0.0001	0.0001	0.0001	0.0134
Classes (man,rem)	Se	0.68	0.64	0.68	0.64	0.61	0.60	0.61	0.56
	Sp	0.47	0.53	0.53	0.60	0.61	0.59	0.54	0.60
	Acc	0.50	0.54	0.54	0.60	0.61	0.59	0.55	0.60
	*p*	0.0605	0.0001	0.0001	0.0001	0.0001	0.0040	0.0659	0.4355

**Table 7:** Results obtained using a single epoch of 1000 samples from each record. A different optimal parameter configuration was found, but performance was fairly similar to previous cases.

	Classes (dep,man)	Classes (dep,rem)	Classes (man,rem)
*m*	5	5	4
γ	0.75	0.75	0.9
Se	0.75	0.68	0.75
Sp	0.69	0.66	0.61
Acc	0.73	0.67	0.62
*p*	**0.0076**	**0.0007**	**0.0432**
MCC	0.4014	0.2939	0.2198

**Table 8:** Results of the Leave-One-Out (LOO) classification evaluation in terms of average accuracy and standard deviation.

Classes (dep,man)	Classes (dep,rem)	Classes (man,rem)
0.75±0.0287	0.58±0.0407	0.62±0.0370

## Appendix A. Slopen Source Code

**function** [pe, Psi_Patt, counter] = SlopeEn(data, dim, gamma, delta)

  **if** nargin < 1, error(’The data time-series must be included’), **end**

  **if** nargin < 2, dim = 6; **end**

  **if** nargin < 3, gamma = 0.94; **end**

  **if** nargin < 4, delta = 0.001; **end**

  n_dp = length(data);

  **if** n_dp <= dim || n_dp < 10, error(’The signal is too short’), **end**

  **if** delta < 1e-12, error(’The delta parameter is too small choose number higher than 1e-12’), **end**

  **if** delta >= gamma, error(’The delta parameter has to be larger than the gamma parameter’), **end**

  Psi_Patt = [];

  counter = [];

  patt = nan(1,dim-1);

  pe = 0;

  **for** k = 1:n_dp-(dim-1)

    subsec = data(k:k+dim-1);

    lg_gamma = subsec(2:end) > (subsec(1:end-1)+gamma);

    lg_delta = subsec(2:end) > (subsec(1:end-1)+delta);

    lg_ab_delta = abs(subsec(2:end) - subsec(1:end-1)) <= delta;

    lg_n_gamma = subsec(2:end) < (subsec(1:end-1)-gamma);

    **for** m = 1:dim-1

      **if** lg_gamma(m)

        patt(m) = 2;

      **elseif** lg_delta(m)

        patt(m) = 1;

      **elseif** lg_ab_delta(m)

        patt(m) = 0;

      **elseif** lg_n_gamma(m)

        patt(m) = -2;

      **else**

        patt(m) = -1;

      **end**

    **end**

    n_patt = length(counter);

    act_match = false;

    **for** m = 1:n_patt

      **if** sum(Psi_Patt(m,:) == patt) == dim-1

        counter(m) = counter(m)+1;

        act_match = true;

        **break**

      **end**

    **end**

    **if** act_match

      Psi_Patt = [Psi_Patt; patt];

      counter = [counter; 1];

    **end**

  **end**

  n_patt = length(counter);

  **for** k = 1:n_patt

    p = counter(k)/n_patt;

    pe = pe + (-p*log2(p));

  **end**

**end**
